# Performance of Epidemic Preparedness and Response Committees to Disease Outbreaks in Arua District, West Nile Region

**DOI:** 10.1155/2019/1437920

**Published:** 2019-02-03

**Authors:** Robert Afayo, Muzamil Buga, John Bosco Alege, Pardon Akugizibwe, Christine Atuhairwe, Ivan Mugisha Taremwa

**Affiliations:** Clarke International University, P.O. Box 7782, Kampala, Uganda

## Abstract

The Epidemic Preparedness and Response Committees (EPPRCs) are at the heart of preventing outbreaks from becoming epidemics by controlling the spread. Evidence-based information regarding factors associated with the performance of EPPRCs in preparedness and response to disease outbreaks is needed in order to improve their performance. A cross-sectional study involving 103 EPPRC members was carried out in Arua district, West Nile region, between the months of July and December 2014. Data were collected using a structured questionnaire, and the chi-square test was used to establish associations. Forty-eight percentage of EPPRC members showed a moderate level of preparedness, and only 39.8% of them had a moderate level of response. The performance drivers of preparedness and response were dependent on presence of a budget (*χ*2 = 10.281, *p*=0.002), availability of funds (*χ*2 = 5.508, *p*=0.019), adequacy of funds, (*χ*2 = 11.211, *p*=0.008), support given by health development partners (*χ*2 = 19.497, *p*=0.001), and motivation (*χ*2 = 20.065, *p* < 0.001). Further, membership duration (*χ*2 = 13.776, *p*=0.001) and respondent cadre (*χ*2 = 12.538, *p*=0.005) had a significant association. Based on these findings, there is a big gap in the preparedness and response ability, all of which are dependent on the financial gap to the Committees. To this, funding for preparedness and response is a critical aspect to respond and contain an outbreak.

## 1. Introduction

**Figure 1:**
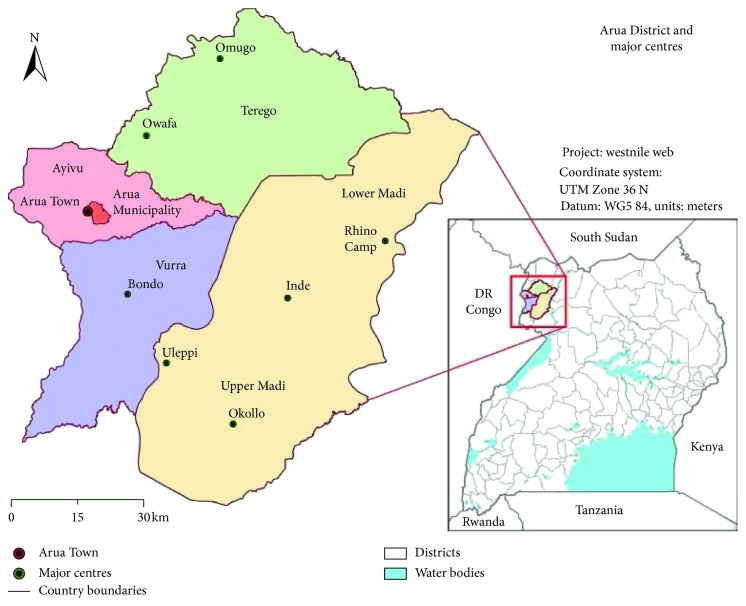
Adopted from https://www.google.com/url?sa=i&source=images&cd=&cad=rja&uact=8&ved=2ahUKEwizi9Hz67feAhXMyYUKHfSACcQjRx6BAgBEAU&url=https%3A%2F%2Fbmcpublichealth.biomedcentral.com%2Farticles%2F10.1186%2Fs12889-017-4589-9&psig=AOvVaw2Hk7L_5ZwKBVO_sm6o1aGP&ust=1541321421556437 (accessed on November 2, 2018).

Arua district in Uganda is one of the districts that has experienced more outbreaks in the country, with 4 cholera outbreaks and a meningococcal meningitis outbreak reported and confirmed in the 2013/2014 financial year alone [1]. The recent outbreaks of meningococcal meningitis in West Nile highlighted the gaps in public health vigilance: Adjumani district which reported the index case had 53 cases and 1 death while Arua had 47 cases and 3 deaths. The Arua district had a case fatality rate (CFR) that was 5 times that of Adjumani, suggesting gaps that may be related to preparedness and response to outbreaks [2]. In the last 5 years, the District Health Office of Arua reactivated Epidemic Preparedness and Response Committees (EPPRCs) at the district and subcounty levels to coordinate the activities of prevention, preparedness and response to disease outbreaks; health development partners (HDPs) such as the United Nations International Children's Emergency Fund (UNICEF) have also provided trainings for the committees. However, not much change has been observed in reducing the frequency and case fatalities in outbreaks and epidemics [1]. The problem is further compounded by the free movement between Arua and the neighboring Democratic Republic of the Congo, a country with a less vigilant public health system [1]. Despite being prone to outbreaks, EPPRC performance and preparedness in the district has not been explored. This research sought to assess the performance of EPPRC members in preparedness and response to disease outbreaks in the Arua district as well as identify factors that may affect their performance (Figure 1).

## 2. Methods

### 2.1. Study Population

The categories of respondents were male and female members of the District Epidemic Prevention Preparedness Committee (DEPPRC) and all the Subcounty Epidemic Prevention, Preparedness and Response Committees (SEPPRCs).

### 2.2. Design and Sampling Procedures

A descriptive cross-sectional study design was adopted to capture data from members of DEPPRC and all the SEPPRCs using a structured questionnaire. This study was carried out between the months of July and December 2014. Using the Krejcie and Morgan formula [3], a sample of 103 respondents was considered adequate for the study.

### 2.3. Measuring Preparedness and Response

Using the Integrated Disease Surveillance and Response (IDSR) guideline, a check list (Appendix) of 15 measures was used to assess the level of preparedness of respondents in different locations with yes and no indicating presence and no presence to the measure, respectively. Similarly, response was measured using the IDSR guideline check list, with yes and no indicating compliance and none compliance to the measure, respectively. At the end of the 15 standard questions asked to measure the level of preparedness/response, a scale was developed to measure the overall level of preparedness/response in disease outbreaks with those scoring 0–5 as low, 5–10 as moderate, and 11–15 as high level.

### 2.4. Measuring Performance

At the end of every questionnaire, the overall performance of EPPRC members in preparedness and response to disease outbreaks was assessed by compiling the level of preparedness and response to make a total of 30 as the highest and 0 as the lowest performance. A score of 0–15 was rated as low performance and above 15–30 as high performance. The bulk (65/103) 63% of respondents had a low performance in preparedness and response to disease outbreaks.

### 2.5. Ethical Considerations

The study obtained ethical approval from Clarke International University (formerly, International Health Sciences University), further permission was sought and obtained from the Arua District Health Office, and EPPRC members provided consent to participate in the study.

## 3. Results

A total of 93 respondents were interviewed giving a response rate of 86% at the subcounty level. While 8 out of 10 were interviewed at the district level, giving a response rate of 80%. The overall response rate for the study is therefore 83%.

As shown in Table 1, majority 82 (79.61%) of the respondents were males and about half were aged 36–50 years. Forty-eight percent of the participants had served as committee members for 2–5 years. Of the 103 respondents interviewed, 37 (35.92%) were political leaders while 35 (33.98%) were health workers and 23 (22.33%) belonged to the category of environmental staff.

### 3.1. Overall Level of Preparedness for Disease Outbreak

**Figure 2:**
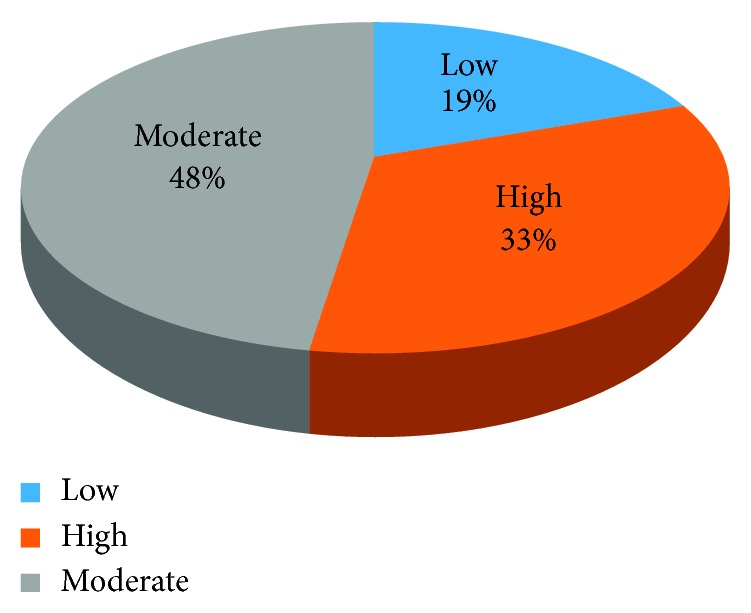
Overall level of preparedness for disease outbreak.

At the end of the 15 standard questions asked to measure the level of preparedness, a scale was developed to measure the overall level of preparedness in disease outbreaks with those scoring 0–5 as low, 5–10 as moderate, and 11–15 as high level. The results are summarized in Figure 2.

### 3.2. Level of Preparedness for Disease Outbreak in Arua District

Majority of respondents (85 (82.5%)) acknowledged the presence of EPPRCs in their localities. Sixty percent of respondents stated that the roles and responsibilities of committee members were clearly stated. Only 38 (36.9%) of participants said there were available stock piles of emergency supplies. Details are presented in Table 2.

Of the 103 respondents, 71 (68.9%) of the respondents knew of the surveillance system available to track and detect disease outbreaks. In addition, 71 (68.9%) had an outbreak treatment centres available in their subcounties. About half of the respondents, 51 (49.5%) used maps of water sources, food stalls, and markets to prevent the spread of an outbreak. Most of the respondents (75 (72.8%)) reported a clear established referral system for patients in case of an outbreak. Sixty six (64.1%) reported that training was offered to them on preparedness to outbreaks, 58 (56.3%) agreed that isolation facilities in their subcounties were available, and 66 (64.1%) of the respondents said that there were laboratories to confirm cases of common outbreaks. About half (53 (51.5%)) were not aware of the protocol for investigating outbreaks. The majority of respondents (95 (92.2%)) had no meetings of EPPR to prepare for an outbreak. At the end of the 15 standard questions asked to measure the level of preparedness, the preparedness score is summarized in Figure 2.

### 3.3. Level of Response

Accordingly, 53 (51.5%) of the respondents agreed that their roles in response to outbreaks were clearly stated. About half (55 (53.4%)) of the respondents said precautions were taken by their committees to prevent spread of a disease during an outbreak. Forty nine (47.6%) adhered to the outbreak response plan. Eighty seven (81.6%) agreed that sanitation promotions were carried out during disease outbreaks. In addition, raising community awareness during outbreaks was reported by 84 (81.6%), but 57 (55.3%) of the participants stated that notification of higher authorities during the previous outbreak was not timely. Five in ten of respondents also reported that response by Ministry of Health (MoH) was equally untimely in the last outbreak and 56 (54.4%) of the respondents adhered to the response plan. Half of the participants (52 (50.4%)) were provided with soap in cholera outbreaks, and majority of the respondents (62 (60.2%)) made use of surveillance data to respond to an outbreak.

Also, 52 (50.5%) of the respondents reported absence of rapid response teams in their committees, and 65 (63.1%) of the respondents reported that the meetings during outbreaks were not frequent. Overall, a larger proportion reported a moderate level of response to disease outbreaks in Arua district, Northern Uganda, are summarized in Table 3.

### 3.4. EPPRCs Factors Associated with Performance in Preparedness and Response to Disease Outbreaks in Arua, Northern Uganda

EPPRC membership duration (*χ*2 = 13.776, *p*=0.001) and cadre of EPPRC (*χ*2 = 12.538, *p*=0.005) were found to be significantly associated with the performance of EPPRCs in preparedness and response to disease outbreaks in Arua, as shown in Table 4.

### 3.5. Performance Drivers of Preparedness and Response to Disease Outbreaks in Arua District

Apart from knowledge on common outbreaks and time of notification, all other factors were found to be significantly associated with performance in outbreak preparedness and response (Table 5). Therefore, knowledge on common outbreaks and time of notification to the MoH were not performance drivers of preparedness and response to disease outbreaks in Arua district, Northern Uganda.

## 4. Discussion

### 4.1. Level of Preparedness

This study found that one out of every three EPPRC members scored moderately in the level of preparedness. This is explained by the fact that much as the EPPRC members were highly motivated to play their roles and were doing so in certain aspects but were at the same time limited by other factors such as financial and logistical insufficiencies. In another study carried out to measure the level of disaster preparedness of San Francisco's community- and faith-based organizations, over 90% were found to have low levels of preparedness [3]. Another study evaluated different countries for their level of preparedness to a possible influenza outbreak. Sixty-two percent of the countries were found to have low levels of preparedness [4]. The difference in the results may be due to the different measures used. This study has established that more than three-quarters of all subcounties in Arua had the EPPRCs as an intervention to the frequent outbreaks. In every 10 EPPRC members interviewed, about 6 had undergone training in preparedness and response to disease outbreaks. Half of EPPRCs in the study had disaster preparedness plans. This was due to the fact that only active EPPRCs were in a position to make preparedness plans as others waited for the disaster to strike before making the plans. Although plans are only one element in overall preparedness, they do constitute a very important element. First responders, emergency planners, and disaster researchers all contend that emergency operation plans should be derived from careful analysis of the types of hazards to which a community is vulnerable [5]. Average level of preparedness to outbreaks by the EPPRC members is not good enough, especially in outbreak prone areas such as Arua district, and should be one of the reasons for the spread of diseases since a well-prepared EPPRC member helps to prevent spread, hence reducing the cases and in some instances preventing the outbreak from reaching a location.

### 4.2. Level of Response

On rating the level of response of EPPRC members, this research found it as moderate. Despite performing highly in community awareness raising, use of surveillance data, the EPPRCs were let down by failure to have frequent meetings during outbreaks, low sanitation promotion among others. The moderate level of response in this study was higher compared to a study carried on 900 social services and emergency management organizations to identify the level of response to an emergency where 9 out of 10 had a low level of response [6]. The study of Gillespie and Steeter had a sample size of 900 organizations compared to this study whose sample size is much smaller leading to a difference in the results because if the sample size of this study 36 is further increased, the mean level of response will be skewed to the left leading to a low score in response [6]. Contrary to this study, the annual health sector performance report of 2012/13 states a 2-fold increase in the frequency of committee meetings in the districts across the country during disease out breaks MoH [1]. The reason for the low level of isolation may be attributed to insufficiency in infrastructure for isolation. Contrary to the observed findings, a study to determine preparedness to bird flu found that most respondents recognized isolation as a vital strategy to prevent the spread of bird flu although they cited the need for social interactions as a challenge in implementing isolation [4]. Low level of isolation leads to the further spread of an infectious disease resulting in mostly a higher number of cases which if uncontrolled will further end up into an epidemic with high morbidity and case fatality. The EPPRC members play a crucial role in creating community awareness and information dissemination, and this role is well played as the results show that three-quarters of the respondents said they played a role in raising community awareness. The high score in raising community awareness was because most of the EPPRC members understood their role in raising awareness of an outbreak in the communities they serve. The World Health report recommends that information must be made public of a behaviour that might reduce the risk of an outbreak [7]. The guideline goes ahead to emphasize publically that this information can have a possibility of changing over time [7].

### 4.3. Performance in Preparedness and Response

Generally, performance in preparedness and response was low with more than 6 out of 10 respondents performing poorly. We attributed the poor performance to lack of knowledge of the roles and responsibilities of the EPPRC members, in adequate funding and the perception that disease outbreaks preparedness and response is the responsibility of health workers alone. Low performance in the preparedness and response was also cited in the WHO report resulting in over 730,361 cases of cholera and 10% case fatality rate in Africa [7].

### 4.4. Performance Drivers on Preparedness and Response to Outbreaks

This study found out that EPPRC members were knowledgeable about outbreaks in Arua. The knowledge of the EPPRCs was high because of the frequency in outbreaks in the district in the last 10 years. Although there was no statistical justification of a relationship between knowledge and performance in this study, it is likely that performance was as a result of limited funding [5]. During the study, no EPPRC in any location was found with funds ready to respond to an outbreak. To this effect, although the Uganda Government allocated at least 1.5% of its total annual budget for disaster preparedness and response in disaster management policy [1], this may be insufficient to meet the ever-increasing epidemic outbreaks. The high motivation in the EPPRCs is sparked by the fact that most of the members interviewed see their role in the committee as crucial in prevention, preparedness and response to outbreaks which have recently been common in the district [8]. To this, it is worthy to scale up prevention strategies during the assessment and management of the outbreak instead of concentrating on only the medical aspects of containing the disease.

### 4.5. Demographic Factors Associated with Performance in Preparedness and Response

The study revealed that more than three-quarters of the EPPRC members were male although no association was found between gender and performance in preparedness and response. This is similar to what was found in a previous study that the field of emergency management is a male-gendered occupation [5]. On the other hand, our findings contravene a report that showed a significant association of gender with performance in emergency preparedness and response [9]. Further, the duration served as a member of the EPPRC means experience, where the longer the duration the more the experience and therefore better performance in preparedness and response. This is similar to what was reported in Northern China [10].

## 5. Conclusion

The performance of the EPPRC members in preparedness and response to outbreaks was wanting, and this calls for concerted efforts to improve training and resource availability as Uganda grapples with regular epidemic outbreaks. The locality of Arua is unique in its geographical neighbourhoods, and also, sanitation challenges as living conditions were demised by the long-time Lord's Resistance Army (LRA) insurgence. Further, there is growing need to foster collaboration with Health Development Partners (HDPs) so as to improve the performance of EPPRC members in preparedness and response to outbreaks.

## Figures and Tables

**Table 1:** Demographic characteristics of the respondents.

Variable	Number (percentage)
*Sex*	
Male	82 (79.61)
Female	21 (20.39)

*Age group*	
20–35 years	45 (44.12)
36–50 years	50 (49.02)
55–70 years	7 (6.86)

*Duration as a member*	
<2 years	24 (24.00)
2–5 years	48 (48.00)
>5 years	28 (28.00)

*Cadre*	
Political leader	37 (35.92)
Health worker	35 (33.98)
Environmentalist	23 (22.33)
Others	8 (7.77)

**Table 2:** Level of preparedness to disease outbreaks.

Variables	Number (percentage)
*Presence of EPPRC*	
Yes	85 (82.5)
No	18 (17.5)

*Clarity or roles of members*	
Yes	67 (65.0)
No	36 (35.0)

*Availability of stock of supplies*	
Yes	38 (36.9)
No	65 (63.1)

*Presence of plans for action*	
Yes	52 (50.5)
No	51 (49.5)

*Presence of volunteers and peripheral health staff*	
Yes	76 (73.8)
No	27 (26.2)

*Availability of surveillance systems*	
Yes	71 (68.9)
No	32 (31.1)

*Treatment centres for outbreaks in locality*	
Yes	71 (68.9)
No	32 (31.1)

*Map of water sources and food stalls*	
Yes	51 (49.5)
No	52 (50.5)

*Transport routes in and out of the area*	
Yes	62 (60.2)
No	26 (25.5)

*Established referral systems*	
Yes	75 (72.8)
No	28 (27.2)

*Level of training in outbreaks*	
Yes	66 (64.1)
No	37 (35.9)

*Sites for isolation*	
Yes	58 (56.3)
No	45 (43.7)

*Labs to confirm cases*	
Yes	66 (64.1)
No	37 (35.9)

*Protocol for investigating outbreaks*	
Yes	50 (48.5)
No	53 (51.5)

*Frequency of meetings before outbreaks*	
Yes	8 (7.8)
No	95 (92.2)

**Table 3:** The level of response to disease outbreaks in Arua.

Variable	Number (percentage)
*Clarity of response roles of committee members*	
Yes	53 (51.5)
No	50 (48.5)

*Limitations of threat by taking precautions*	
Yes	55 (53.4)
No	48 (46.6)

*Level of adherence to response plans*	
Yes	49 (47.6)
No	54 (52.4)

*Further training and use of volunteers in case detention*	
Yes	49 (47.6)
No	54 (52.4)

*Cases are isolated in shelters during outbreaks*	
Yes	48 (46.6)
No	55 (53.4)

*Supply of safe drinking water during cholera outbreaks*	
Yes	44 (42.7)
No	87 (81.6)

*Sanitation promotion during outbreaks*	
Yes	87 (81.6)
No	19 (18.4)

*Raising community awareness*	
Yes	84 (81.6)
No	19 (18.4)

*Timeliness of notification of MoH*	
Yes	46 (44.7)
No	57 (55.3)

*Response timeliness of authorities*	
Yes	54 (52.4)
No	49 (47.6)

*Level of adherence to response plans*	
Yes	56 (54.4)
No	47 (45.2)

*Provision of soap in cholera outbreaks*	
Yes	52 (50.5)
No	51 (49.5)

*Use of surveillance data for outbreak response*	
Yes	62 (60.2)
No	41 (39.8)

*Presence of RRT*	
Yes	51 (49.5)
No	52 (50.5)

*Frequency of meeting during outbreak*	
Yes	38 (36.5)
No	65 (63.1)

**Table 4:** Demographic factors of the EPPRCS in preparedness and response to disease outbreaks.

Variable	Number (%)	High performance	Low performance	*χ*2	*p* value
*Sex*					
Male	82 (79.6)	33 (86.8)	49 (75.38)	1.940	0.062
Female	21 (20.4)	5 (13.2)	16 (24.62)		

*Age*					
20–35 years	45 (44.1)	22 (59.5)	23 (35.4)	5.561	0.062
36–50 years	50 (49.0)	13 (35.1)	37 (56.9)		
55–70 years	7 (6.9)	2 (5.4)	5 (7.7)		

*Duration as member*					
<2 years	24 (24.0)	5 (13.9)	19 (29.7)	13.78	0.001
2–5 years	48 (48.0)	13 (36.1)	35 (54.7)		
>5 years	28 (28.0)	18 (50.0)	10 (15.6)		

*Cadre*					
Political leader	37 (35.9)	12 (31.6)	25 (38.46)	12.538	0.005
Health worker	35 (34.0)	7 (18.4)	28 (73.1)		
Environmentalist	23 (22.3)	14 (36.8)	9 (13.9)		
Others	8 (7.7)	5 (13.2)	3 (4.6)		

*p* value < 0.05 is statistically significant.

**Table 5:** Performance drivers of the EPPRCs in outbreak preparedness and response in Arua district.

Variable	Number (%)	High performance	Low performance	*χ*2	*p* value
*Knowledge of common outbreaks*					
Measles	5 (4.85)	2 (5.26)	3 (4.62)	3.298	0.366
TB	10 (9.71)	2 (5.26)	8 (18.31)		
Meningitis	7 (6.80)	1 (2.63)	6 (9.23)		
Cholera	81 (78.64)	33 (86.84)	48 (73.35)		

*MoH should be notified within*					
Within 24 hours	85 (84.16)	33 (91.67)	52 (80.00)	2.365	0.124
More than 24 hours	16 (15.84)	3 (8.33)	13 (20.00)		

*Have a budget*					
Yes	44 (42.72)	24 (63.16)	20 (30.77)	10.281	0.002
No	59 (57.28)	14 (36.84)	45 (69.23)		

*Availability of funds*					
Yes	14 (13.73)	9 (24.32)	5 (7.69)	5.508	0.019
No	88 (86.27)	28 (75.68)	60 (92.31)		

*Have support*					
Yes	61 (59.22)	30 (78.95)	31 (37.69)	9.701	0.008
No	42 (40.78)	8 (21.05)	34 (52.31)		

*Financial support adequate*					
Strongly agree	6 (5.83)	1 (2.63)	5 (7.69)	11.211	0.001
Agree	17 (16.5)	7 (18.42)	10 (15.38)		
Disagree	46 (44.66)	24 (63.16)	22 (33.85)		
Strongly disagree	34 (33.01)	6 (15.79)	28 (43.08)		

*Support form given by HDP*					
Supplies	33 (34.74)	18 (52.94)	15 (24.59)	19.497	<0.001
Technical support	16 (16.84)	8 (23.53)	8 (13.11)		
Human resource	13 (13.68)	4 (11.76)	9 (14.75)		
Financial support	10 (10.53)	4 (11.76)	6 (9.84)		
No support	23 (24.21)	0 (0.00)	23 (37.7)		

*HDPs are very helpful*					
Yes	55 (56.7)	30 (85.71)	25 (40.32)	18.775	<0.001
No	42 (43.3)	5 (14.29)	37 (59.68)		

*Sufficiently facilitated*					
Strongly agree	14 (13.86)	8 (21.05)	6 (9.52)	14.504	0.002
Agree	34 (33.66)	16 (42.11)	18 (28.57)		
Disagree	35 (34.65)	14 (36.84)	21 (33.3)		
Strongly disagree	18 (17382)	0 (0.00)	18 (28.57)		

*I am making a contribution*					
Strongly agree	38 (37.62)	23 (60.53)	15 (23.81)	15.229	0.002
Agree	45 (44.55)	12 (31.58)	33 (52.38)		
Disagree	12 (11.88)	3 (7.89)	9 (14.29)		
Strongly disagree	6 (5.94)	0 (0.00)	6 (9.52)		

*Feel encouraged to perform duties*					
Strongly agree	32 (31.68)	21 (55.26)	11 (17.46)	20.065	<0.001
Agree	46 (45.54)	15 (39.47)	31 (49.21)		
Disagree	11 (10.89)	0 (0.00)	11 (17.46)		
Strongly disagree	12 (11.88)	2 (5.26)	10 (15.87)		

*Role recognized by community*					
Yes	83 (81.37)	37 (97.37)	46 (71.88)	10.223	0.001
No	19 (18.63)	1 (2.63)	18 (28.13)		

*p* value < 0.05 is statistically significant.

## Data Availability

The data used to support the findings of this study are available from the corresponding author upon request.

## Appendix

### Quantitative Questionnaire

#### Section A: Background Information

Questionnaire number…………………………..

1. Sex: 1. □ Male 2. □ Female
2. Age (a) Below 20 (b) 20–35 (c) 36–50 (d) 55–70 (e) Above 70
3. Duration as a member (a) Below 2 years (b) 2–5 years (c) above 5 years
4. Location/Sub county…………………………
5. Position/Cadre (a) Political leader (b) health worker (c) Environmental staff (d) Others specify..............

#### Section B. Level of Preparedness

**Table:**

No.	Outbreak Preparedness Questions (*ANSWER YES OR NO*)

1	Do you have an EPPRC?
2	Are their established roles and responsibilities for members?
3	Established plan for action for tackling disease outbreaks
4	Availability of stock of essential supplies and inter agency emergency kits for common outbreaks
5	Availability of volunteers and peripheral health staff
6	Do you have surveillance systems in place
7	Availability of treatment centre for outbreak in the locality
8	Map of water sources, food stalls, sanitation, slaughter houses And transport routes in and out of the area
9	Established referral system
10	Health care staff trained in preparedness and response
11	Provision of health education to the community(awareness)
12	Presence of shelter for site of isolation
13	Laboratory is identified for confirmation (locally, regionally or nationally)
14	Protocol for investigation an outbreak
15	Does the committee meet frequently

#### Section C. Level of Response

**Table:**

No.	Outbreak Response Question(*ANSWER YES OR NO*)

1	Clarity of the roles and responsibilities of members
2	Limit the spread, take precaution in neighboring areas, check movement in and out of area
3	Is the outbreak response plan being followed in the area
4	Further training and use of volunteers in case detection
5	Cases were isolated in shelters
6	Supply of safe water and sufficient water in case of cholera out break
7	Sanitation promotion like latrines and waste management was done
8	Community awareness about outbreak was raised
9	Ministry of health was notified within 24 hours
10	Authorities responded within 48 hours of outbreak
11	Protocol for investigating outbreak was implemented.
12	Provision of soap in cholera outbreaks
13	Surveillance data was used to detect and monitor outbreak control.
14	Is there a rapid response team in your area
15	Do the members meet at least weekly during an outbreak

#### Section D. Drivers of Performance

1. Knowledge
  - What is a disease outbreak? .....................................................................................
  - What is the commonest epidemic in Arua in the last 3 years? □ Measles □ TB □ Meningitis □ Cholera
  - What is the recommended time within which the MoH should be notified in case of an outbreak? □ 20 hours □ 24 hours □ Meningitis 48 hours □ 72 hours
  - What is the recommended time within which MoH should respond in case of an outbreak? □ 20 hours □ 24 hours □ Meningitis 48 hours □ 72 hours
2. Funding
  - Do you have a Budget for preparedness and response to disease outbreaks? □ Yes □ No
  - Are funds available to the committee in to immediately prepare and response to an outbreak? □ Yes □ No
  - The funds for preparedness and response in my area is adequate □ Strongly agree 2. □ Agree □ Disagree 2. □ Strongly disagree
3. Support of the Health Development Partners (HDP)
  - Do you have (HDP) supporting disease outbreak preparedness and response? □ Yes □ No
  - What is the form of support given is given by the HDPs? □ Supplies □ Technical support □ Human resource □ Financial support □ No support is offered □ Others, Specify………………......
  - The HDPs are very helpful in my area in preparedness and response to disease outbreaks □ Yes □ No
4. Motivation
  - I am you sufficiently facilitated to perform my duties as a member of EPPRC? □ Strongly agree 2. □ Agree □ Disagree 2. □ Strongly disagree
  - I am making a big contribution in the preparedness and response to disease outbreaks in my community? □ Strongly agree 2. □ Agree □ Disagree 2. □ Strongly disagree
  - I feel personally encouraged to perform my duties as a member of EPPRC □ Strongly agree 2. □ Agree □ Disagree 2. □ Strongly disagree
  - The role I play as a committee member is recognized by my community? □ Yes □ No

## Additional Points

The findings of the study must be considered with caution because systemic error may have influenced results differently. Selection bias could have occurred where politicians could have overrated the level of preparedness and response resulting in an overestimated performance. Random error could have influenced the results of this research either by overestimating or underestimating the findings. The results of this study might only be generalized for the Arua district because of its unique frequency to disease outbreaks and epidemics compared to other parts of the country. Another limitation of the study is that multivariate analysis was not conducted as it was deemed not practical. As such, we were unable to identify significant variables that were associated with outcome measures of interest.
